# Bile acid sequestration reveals microbiome remodeling that dominates FXR signaling to shape host responses to enteric infection

**DOI:** 10.1371/journal.ppat.1014558

**Published:** 2026-09-08

**Authors:** Anahita Mansouripour, Drew R. Seeger, Meredith Parmer, Peddanna Kotha, Anton M. Golovko, Mikhail Y. Golovko, Shahram Solaymani-Mohammadi

**Affiliations:** 1 Department of Biomedical Sciences, School of Medicine and Health Sciences, University of North Dakota, Grand Forks, North Dakota, United States of America; 2 Laboratory of Mucosal Immunology, Department of Biomedical Sciences, School of Medicine and Health Sciences, University of North Dakota, Grand Forks, North Dakota, United States of America; University of Virginia, UNITED STATES OF AMERICA

## Abstract

Approximately 90 million American adults are hypercholesterolemic, with an estimated 43 million individuals either already using or eligible to receive cholesterol-lowering medications, such as bile acid sequestrants. Although bile acid sequestrants have demonstrated therapeutic efficacy in lowering cholesterol levels in individuals with hypercholesterolemia, their impact on intestinal homeostasis in healthy individuals and those with enteric microbial infections remains unclear. We set out to investigate the potential effects of bile acid sequestration on mucosal immune responses and to evaluate how enteric microbial pathogens colonize the small intestine of mice *in vivo*. We also examined how bile acid sequestration affected intestinal microbial ecosystems before and during enteric infection. We found that bile acid sequestration increased resistance to enteric infection, and this phenotype required farnesoid X receptor (FXR) in myeloid cells. We observed significantly increased diversity and richness in the gut microbiome of mice that were administered a bile acid sequestrant that correlated with enhanced resistance to enteric infection, suggesting that bile acids may function as limiting factors for the diversity and richness of the gut microbiome in health and disease. Notably, the antibiotic-driven depletion of the gut microbiome led to significantly increased susceptibility to infection with both *Giardia duodenalis* and *Giardia muris*. Our findings reveal a previously overlooked role for bile acid sequestrants in the regulation of the gut microbiome as well as host resistance to enteric infections. Bile acid sequestration and the subsequent blockade of pathways downstream of bile acid signaling may modulate host immunity during enteric infections, potentially leading to an altered gut microbiome in individuals taking bile acid sequestrants to lower blood cholesterol.

## Author summary

Human giardiasis is a common enteric parasitic infection that causes millions of infections globally, yet the mechanisms underlying effective parasite clearance are poorly understood. Bile acids act as key signaling molecules that shape the gut microbiome and influence immune responses. Millions of individuals with hypercholesterolemia take bile acid sequestrants to lower cholesterol, but it is still unclear how these medications affect susceptibility to intestinal infections. In the current study, we examined how bile acid sequestration modulates host defenses during *Giardia* infection. We found that bile acid sequestration increased the diversity of the gut microbiome and enhanced early resistance to infection in a murine model of human giardiasis. This protective effect required signaling through the bile acid receptor FXR, but not TGR5, in myeloid cells. Depleting the microbiome with an antibiotic cocktail abolished protection and made mice highly susceptible to infection, indicating that the microbiome plays a dominant role in controlling intestinal *Giardia* colonization. We also observed robust intestinal mast cell activation and increased inflammatory lipid mediators following infection. Together, our findings demonstrate that bile acids regulate intestinal immunity mainly by reshaping the gut microbiome. Understanding how bile acids interact with the microbiome may help guide new strategies to strengthen mucosal defenses against enteric pathogens.

## Introduction

Hypercholesterolemia is a global health concern affecting more than 90 million adults in the United States alone [1,2]. Approximately 43 million Americans either use or qualify for cholesterol-lowering medications [1–4]. As the end-products of cholesterol catabolism, bile acids are biosynthesized in the liver and released into the proximal portions of the small intestine, where they play critical roles in dietary fat digestion and absorption [5,6]. Most bile acids are reabsorbed in the terminal ileum before re-entering the liver through the enterohepatic circulation [5,6].

In addition to their digestive functions, bile acids act as signaling molecules that regulate immune responses and gut microbiome composition through interactions with nuclear receptors (e.g., FXR; farnesoid X receptor*; Nr1h4*; nuclear receptor subfamily 1 group H member 4) and membrane-bound receptors (e.g., TGR5; Takeda G protein-coupled receptor; *Gpbar1*; G protein-coupled bile acid receptor 1) [7–10]. Resident intestinal microbial communities metabolize and convert primary bile acids to secondary bile acids [11,12]. Bile acids modulate the differentiation and function of multiple T cell subsets, as well as the balance between pro- and anti-inflammatory immune responses [13–16]. Secondary bile acids maintain intestinal mucosal immune homeostasis by promoting the generation of FoxP3-expressing regulatory T cells in the colon [16]. They also can inhibit the differentiation of proinflammatory T_H17_ cells by interacting with RORγt, a key transcription factor required for the generation and development of T_H17_ cells [15].

Bile acid sequestrants have long been used as cholesterol-lowering medications, particularly in patients with hypercholesterolemia who are intolerant to statins [17–20]. Despite their primary use as cholesterol-lowering medications, bile acid sequestrants are widely used to treat bile acid-mediated diarrhea; their reported effects on glucose metabolism and insulin sensitivity further broaden the translational versatility of bile acid sequestrants [5,21–23].

Currently, three FDA-approved bile acid sequestrants, including cholestyramine, colestipol, and colesevelam, are routinely used to treat hypercholesterolemia [18]. Bile acid sequestrants such as cholestyramine bind to free luminal bile acids in the intestine and inhibit their reabsorption via the enterohepatic circulation [24,25].

The increased excretion of sequestered bile acids into the feces triggers a compensatory hepatic mechanism to convert more cholesterol molecules into bile acids, thereby reducing blood cholesterol levels [20,24]. As a result, hepatocytes upregulate the low-density lipoprotein (LDL) receptor expression and enhance the clearance of circulating LDL cholesterol from the bloodstream [19]. In addition to their well-characterized absorption via the enterohepatic circulation, reduced luminal bile acids can also lower the ileal fibroblast growth factor-19 (FXR-FGF19) signaling to hepatocytes, thereby reversing the inhibition of cholesterol 7α-hydroxylase (CYP7A1), the rate-limiting enzyme in bile acid production [26,27]. This feedback loop explains the pharmacodynamic basis of intestinal bile acid sequestration by cholestyramine.

Bile acids have attracted significant attention in recent years as key regulators of the host-pathogen interactions at the mucosal surfaces of the intestine [28–29]. Despite the widespread use of bile acid sequestrants, the extent to which these medications impact intestinal homeostasis in health and disease is poorly understood. Using an experimental murine model of the most commonly identified enteric parasitic infection in the United States, human giardiasis, we aimed to assess how bile acid sequestrants affect intestinal microbial ecosystems and thereby influence host resistance to enteric infection. We found that bile acid sequestration significantly increased bacterial diversity and richness, but not intestinal inflammatory parameters, which correlated with greater resistance to enteric infection. Bile acids may function as limiting factors, restricting the diversity of microbial communities in the intestine. A diverse gut microbiome signature is associated with increased protection against a variety of intestinal pathogens, most likely through competition for nutrients and space [30–34]. We and others have found that the antibiotic-driven depletion of the gut microbiome can render mice substantially more susceptible to parasite colonization in a murine model of *G. duodenalis* infection [35–38]. An increase in the diversity and richness of the gut microbiome in the absence of bile acids is likely a determining factor driving enhanced resistance to enteric infection. Use of bile acid sequestrants may predispose individuals to off-target effects, especially when accompanied by enteric infection.

## Materials and methods

### Ethics statement

Laboratory animal studies were performed at the Center for Biomedical Research at the University of North Dakota in compliance with procedures approved by the Institutional Animal Care and Use Committee (protocol numbers: 2007–2, 2008–6, 2308–0049, 2310–0059) of the University of North Dakota in accordance with the National Institutes of Health Guidelines.

### Mice and diets

Age- and sex-matched wild-type mice on a C57BL/6 background were purchased from Jackson Laboratory (Bar Harbor, ME). Mice were randomized into two groups and were fed either a control diet (NIH-31; Dyets Inc., Bethlehem, PA) or an NIH-31 diet containing 2% cholestyramine (Sigma-Aldrich, Saint Louis, MO) *ad libitum* for 7 weeks and during the entire course of *G. muris* and *G. duodenalis* infection. Fresh control and cholestyramine diets were provided every other day. Uninfected (naïve) control mice were fed either a control or cholestyramine diet for 7 weeks as described, without being infected with *Giardia*.

Mice with conditional deletion of the bile acid receptors FXR (*Nr1h4*^Δmye^) and TGR5 (*Gpbar1*^Δmye^) in myeloid cells were generated by crossing *Nr1h4*^flox/flox^ mice (Jackson) and *Gpbar1*^flox/flox^ (Taconic Biosciences, Rensselaer, NY) to mice expressing the myeloid cell-specific promoter (LysM-Cre; Jackson). Successful Cre-mediated genetic recombination was validated by genotyping protocols using genomic DNA isolated from tail snips, followed by confirmation by Western blot analysis using anti-mouse FXR (clone D3; Santa Cruz Biotechnology, Dallas, TX) monoclonal antibody and TGR5 (Abcam) polyclonal antibody. Age- and sex-matched *Nr1h4*^flox/flox^ mice and *Gpbar1*^flox/flox^ littermates (no Cre transgenes) were used as controls.

### Infection protocol and trophozoite quantification

*G. muris* (strain Roberts-Thomson) and *G. duodenalis* (strain H3) cysts were purchased from Waterborne Inc. (New Orleans, LA). Mice were infected with *G. muris* (10^4^ cysts/mouse) or *G. duodenalis* (10^5^ cysts/mouse) in 0.2 mL of sterile PBS (pH 7.4) administered via oral gavage. Mice were euthanized at indicated time points post-infection, and the entire small intestine was removed, opened longitudinally, cut into smaller pieces, and placed in 50 mL Falcon tubes containing sterile PBS, as described [35,36]. Tubes were placed on ice for 30–45 min to detach trophozoites, vortexed vigorously for 30 s, and the motile trophozoites were counted using a hemocytometer. Parasite burdens were expressed as the numbers of trophozoites/small intestine. Intestinal samples from animals with no detectable live *Giardia* trophozoites were assigned a log_10_ value corresponding to one-half the lower limit of detection.

### Antibiotic treatment

To perturb microbial diversity in the intestinal tract, mice were given an antibiotic cocktail containing ampicillin (1 mg/mL; Sigma-Aldrich), neomycin trisulfate (1.4 mg/mL; Sigma-Aldrich), and vancomycin hydrochloride (1 mg/mL; Gold Biotechnology, Saint Louis, MO) in drinking water *ad libitum* for 3 days before and during the entire course of *Giardia* infection [35,36]. Antibiotic solutions were freshly prepared and replaced every other day.

### Aerobic and anaerobic bacterial culture of fecal samples

Fresh fecal samples of experimental and control mice before and after antibiotic treatment were aseptically collected by placing mice in separate clean cages with no bedding. Fecal pellets (100–200 mg) were weighed, homogenized in sterile PBS (pH 7.4), serially diluted 10-fold, plated onto tryptic soy agar (TSA) with 5% sheep blood (Teknova, Hollister, CA), and kept at 37 °C for 24 h. The culturable anaerobic fecal bacteria were grown by plating fecal pellet dilutions on TSA with 5% sheep blood using a BD GasPak EZ anaerobic pouch system (BD, Sparks, MD), then incubated at 37 °C for 72 h. Bacterial numbers were counted and expressed as colony-forming unit (CFU)/g feces.

### Stool antigen detection

A *Giardia*/*Cryptosporidium* QUIK CHEK assay (TechLab, Blacksburg, VA) was used to detect *G. duodenalis* antigens in mice fed either a control or cholestyramine diet following the manufacturer’s instructions [39,40]. Briefly, fecal materials were diluted using the kit diluent, applied to the test cassettes, followed by incubation for 15 min. Cassettes were washed once with 300 µL of the kit’s wash buffer, incubated for an additional 10 min following substrate addition, and the results were subsequently analyzed by eye.

### Quantification of hydroxyeicosatetraenoic acid (HETE) stereoisomers and bile acid profile by targeted ultra-performance liquid chromatography-tandem mass spectrometry (UPLC-MS/MS)

Quantification of HETE stereoisomers and bile acid profiles by targeted UPLC-MS/MS was performed, as previously described [41,42]. Whole small intestines of uninfected and infected mice 7 days after *G. muris* infection were aseptically dissected, opened longitudinally, and cleaned of luminal contents. A small piece of mid-jejunum (1–2 cm) was collected, promptly snap-frozen in liquid nitrogen, and kept at cryogenic temperature prior to HETE extraction. Intestinal tissues were pulverized under liquid nitrogen, and 10 mg of tissue powder was extracted with 90 µL of methanol containing 1 ng of 12(±)-HETEd8 and 5(S)-HETEd8 (Cayman Chemical Co., Ann Arbor, MI) as internal standards.

Tissue samples were sonicated using a sonic dismembrator equipped with a 4C15 probe (Fisher Scientific, Waltham, MA), followed by centrifugation at 10,000 × *g* for 15 min at room temperature according to previously described protocol [41,42].

A 10-µL aliquot was injected into the UPLC-MS/MS system for analysis. UPLC-MS/MS instrumentation and LC conditions were performed, as previously described [41]. Briefly, an Acquity UPLC system (Waters, Milford, MA) equipped with an Acquity UPLC HSS T3 column (1.8 µm, 100 Å pore diameter, 2.1 × 150 mm) with Vanguard precolumn was used. HETE molecular species were resolved with a gradient of acetonitrile and water containing 0.1% formic acid. Xevo TQ-S MS (Waters) was operated in multiple reaction monitoring mode set to negative electrospray ionization. The MS/MS transitions were set at 319.20/115.20 for 5-HETE, 319.20/155.20 for 8-HETE, 319.20/179.10 for 12-HETE; 319.20/219.09 for 15-HETE, 327.12/116.03 for 5-HETE-d8, and 327.12/184.04 for 12-HETE-d8. Instrument control, acquisition, and sample analysis were performed using MassLynx V4.2 software (Waters, Milford, MA).

Mice were anesthetized with isoflurane and blood was collected into ethylenediaminetetraacetic acid (EDTA) tubes. Blood was subjected to centrifugation, and plasma was separated. Twenty µL of plasma was extracted with 60 µL of methanol containing 1 ng of tauro-cholic-d5, 1 ng of cholic-d4, 1 ng of glycocholic-d4, and 10 ng of chenodeoxycholic-d4 (Medical Isotopes Inc., Pelham, NH) as internal standards [42]. For quantifying bile acids in the jejunum, tissues were pulverized under liquid nitrogen and 10 mg of tissue powder was extracted with 90 µL of methanol containing internal standards at the amount described for plasma. Ten µL of plasma or tissue extract was injected into the UPLC-MS/MS system for analysis. UPLC-MS/MS instrumentation and LC conditions, control, acquisition, and sample analysis were performed as described above for HETE analysis. The MS/MS transitions were set at 514.40/123.90 for tauro-cholic, tauro-α-muricholic, tauro-ω-muricholic, and tauro-β-muricholic, 498.32/123.91 for tauro-deoxycholic, 498.29/123.91 for tauro-chenodeoxycholic, 464.30/402.12 for glycocholic, 448.30/386.40 for glycochenodeoxycholic, 407.31/343.18 for cholic, α-muricholic, ω-muricholic, and β-muricholic, 391.29/373.17 for deoxycholic, chenodeoxycholic, and lithocholic, 519.30/123.90 for tauro-cholic-d5, 468.32/406.12 for glycocholic-d4, 411.33/346.96 for cholic-d4, and 395.26/377.30 for chenodeoxycholic-d4, as previously described [41,42].

### Generation of bone marrow-derived macrophages (BMDMs)

BMDMs were generated, as previously described [43,44]. Briefly, the femurs and tibias of age- and sex-matched mice were aseptically removed, bone marrow was flushed out with sterile PBS and the remaining cells were rinsed twice with ice-cold PBS. BMDMs were differentiated by culturing bone marrow-derived progenitors in complete RPMI-1640 supplemented with 30% (v/v) of L929 cell-conditioned medium (ATCC, Manassas, VA) for 1 week. BMDMs were stained with Giemsa (Sigma-Aldrich) to assess morphological characteristics.

### Western blot analysis of FXR and TGR5 expression in BMDMs

Differentiated cells were lysed in ice-cold RIPA buffer (Bioworld, Dublin, OH), supplemented with the Halt Protease/Phosphatase inhibitor cocktail (Thermo Fisher Scientific, Inc.), centrifuged at 14,000 rpm for 5 min to fractionate the supernatant and pellet, followed by measurement of the protein concentration of each sample by the Pierce bicinchoninic acid (BCA) protein assay kit (Thermo Fisher Scientific, Inc., Rockford, IL). Equal amounts of total protein (30 µg) were mixed with the 2 × Laemmli sample buffer (Bio-Rad Laboratories, Hercules, CA), boiled for 5 min, electrophoresed on a 12% acrylamide gel (Bio-Rad), and transferred onto Immobilon-P PVDF membrane (Millipore, Burlington, MA). Non-specific binding was blocked by incubating membranes in 5% BSA (Millipore, Kankakee, IL) in 1 × TBST buffer (Thermo Fisher Scientific, Inc.) for 1 h at room temperature on a horizontal rocker, followed by incubating membranes overnight at 4°C with the mouse monoclonal anti-FXR (1:1000; clone D3; Santa Cruz Biotechnology), rabbit polyclonal anti-TGR5 (1:1000; Abcam), and β-actin (1:1000, Santa Cruz Biotechnology) antibodies. Membranes were washed three times in 1 × TBST buffer before being incubated with peroxidase-conjugated goat anti-rabbit or goat anti-mouse (diluted 1:3000; Jackson ImmunoResearch Laboratories; West Grove, PA) secondary antibodies for 1 h at room temperature. Membranes were washed in 1 × TBST buffer, visualized with enhanced chemiluminescence detection reagent (Clarity Western ECL Substrate, Bio-Rad), and imaged with an Azure C600 digital chemiluminescent imager (Azure Biosystems, Inc., Dublin, CA).

### *Ex vivo* small intestine culture and enzyme-linked immunosorbent assay (ELISA)

The entire mid-jejunum of uninfected and infected mice 7 days after *G. muris* or *G. duodenalis* infection was aseptically removed, and cleaned of feces, fat, and connective tissue. Peyer’s patches were excised, and the intestines were opened longitudinally and washed three times with ice-cold RPMI-1640 medium (Gibco, Bleiswijk, The Netherlands), supplemented with 2 mM L-glutamine, 10% heat-inactivated fetal bovine serum (FBS; R&D Systems, MN), 100 μg/mL penicillin/streptomycin (Gibco, Grand Island, NY), and 50 µM 2-mercaptoethanol [45,46]. Small intestinal tissues were further cut into smaller pieces, and cultured in 700 µL of complete RPMI-1640 medium in 48-well plates (Corning, NY) at 37 °C in a 5% CO2 incubator for 24 h. Culture supernatants were collected, centrifuged at 14,000 rpm for 5 min to remove cellular debris, and assayed for chymase-1, histamine (both from Abbexa, Sugar Land, TX), as well as IL-4, IL-6, IL-17, and IL-23 (DuoSet ELISA kits, all from R&D Systems) per the manufacturer’s instructions. Optical density (OD) was measured at 450 nm using a Synergy HT microplate reader (BioTek Instruments, Inc., Winooski, VT, USA), and results were expressed as picograms of cytokine per gram (pg/g) of small intestine.

### Histopathological evaluation

The entire jejunum was harvested, cleaned, prepared as a Swiss roll, fixed in 10% buffered formalin for 24 h, transferred to 70% ethanol, and embedded in paraffin. Four-micrometer (µm) tissue sections were stained with hematoxylin and eosin (H&E). Histopathological changes were evaluated by a blinded observer using a previously described scoring system [47–49].

### Immunofluorescence staining for mucosal mast cells

Indirect immunofluorescence staining was performed on 4-µm sections of paraffin-embedded jejunal tissues in Swiss roll orientation. Briefly, following deparaffinization and rehydration in sequential alcohols, slides were washed thrice with Tris-buffered saline, then incubated for 15 minutes at room temperature in TBS containing 100 mM glycine, 0.1% Triton X-100, 0.05% Tween 20, and 0.3% hydrogen peroxide. Non-specific antibody binding was blocked with 5% normal goat serum, 1% bovine serum albumin, 1% teleostean gelatin, 100 mM glycine, 0.1% Triton X-100 and 0.05% Tween 20 in Tris-buffered saline for 1 h at room temperature.

Tissue sections were then co-stained with anti-rabbit tryptase (1 µg/mL; MA5–38007, Invitrogen, Waltham, MA) and anti-rat c-Kit/CD117 (2.5 µg/mL; MA1–70079, Invitrogen) monoclonal antibodies at 4°C overnight in TBS containing 100 mM glycine. After thorough washing with TBS, sections were incubated for an additional 1 h at room temperature with Alexa Fluor 488 goat anti-rabbit IgG (1 µg/mL; A11008, Invitrogen) and Alexa Fluor Plus 647 goat anti-rat IgG (1 µg/mL; A48265, Invitrogen) secondary antibodies diluted in TBS-glycine. Nuclei were counterstained with 4,’6-diamidino-2-phenylindole (DAPI, 1 µg/mL; Sigma-Aldrich, D9542) in TBS, and coverslips were mounted using 90% glycerol in 20 mM Tris buffer with 0.5% N-propyl gallate. Matched tissue slides stained with secondary antibody-only (no primary antibody) were prepared in tandem to verify specificity of antibody binding. Images were acquired using an Olympus BX53 upright microscope (Evident Scientific, Waltham, MA), equipped with an Olympus X-Line extended apochromat infinity-corrected 20 × objective and an Olympus DP80 camera (Evident Scientific, Waltham, MA).

### RNA isolation and NanoString analysis

Small pieces of the mid-jejunum (15–30 mg) of uninfected and G. muris infected mice 7 and 14 days after infection were aseptically removed from age- and sex-matched C57BL/6 mice fed a control or cholestyramine diet. Total mRNA was extracted with the automated Promega Maxwell RSC simplyRNA Tissue Kit (Promega, Madison, WI, USA) using a Maxwell RSC instrument AS4500 (Promega) according to the manufacturer’s instructions. Briefly, jejunal tissues were homogenized in 200 µL of ice-cold 1-thioglycerol/homogenization solution, followed by adding 200 µL of the lysis buffer to the homogenates. A tissue sample aliquot (400 µL) was transferred to a Maxwell cartridge, supplemented with 10 µL of DNase I solution per cartridge. Purified RNA samples were eluted in nuclease-free water. RNA concentrations were measured using a Qubit 4 Fluorometer with Qubit RNA Broad Range Assay Kit (Life Technologies), and RNA purity was measured with a NanoDrop (Thermo Fisher Scientific, Inc.). The RNA integrity number for each sample was determined using an Agilent 4200 TapeStation system with RNA ScreenTape (Agilent Technologies, Santa Clara, CA). Total RNA content and purity were determined, and a total of 100 ng of purified RNA from jejunal tissues was used for gene expression analysis using the nCounter Mouse Host Response Panel (NanoString Technologies Inc, Seattle, WA) as described [45,46]. This panel includes 785 genes across 50 pathways, as well as 12 internal control genes to normalize the data. Hybridized samples were initially set up in the nCounter Prep Station (NanoString Technologies Inc) to clean and process the RNA samples, followed by transferring to the nCounter Digital Analyzer, a multi-channel epifluorescence scanner, to acquire the data. The resource compiler (RCC) files were analyzed using the NanoString nSolver analysis software (v3.0). Differential expression was defined as ≥2-fold upregulation with statistical significance (*p* < 0.05). We excluded transcripts that exhibited ≥2-fold induction but did not meet statistical significance.

### Shotgun metagenomic analysis of the fecal microbiome

Mice were placed in separate clean cages with no bedding, and fresh fecal samples were collected aseptically before and after infection. Fecal samples from individual mice (*n* = 4–10/diet/time point) were immediately transferred to barcoded sample collection vials (2 fecal pellets/mouse) in a DNA stabilizing buffer provided by Transnetyx Microbiome (Transnetyx, Inc., Cordova, TN), and shipped for library preparation and whole-genome sequencing. The fecal samples were analyzed to a target depth of 2 million 2 × 150-bp read pairs using the shallow shotgun whole-genome sequencing. Sequencing data were compared against more than 115,000 whole microbial reference genomes using the One Codex microbiome analysis platform (One Codex, San Francisco, CA). The relative abundance of an individual microbial species was approximated based on the depth and coverage of sequencing across all available reference whole-genome data.

### Gene ontology and pathway analysis

Differentially expressed gene ontology enrichment analysis was achieved by a gene panel algorithm provided by NanoString. Normalized NanoString values were used to construct and visualize the heatmaps of differentially expressed genes by using the web-based open tool Morpheus (http://software.broadinstitute.org/morpheus).

### Statistical analysis

Statistical analysis was performed using GraphPad Prism (v9.5.1 for Windows; GraphPad Software, San Diego, CA). A two-tailed unpaired Mann-Whitney *U* test was utilized to determine the statistical differences between the two groups/diets. A one-way ANOVA, followed by Tukey’s post hoc test, was employed for comparing multiple parameters. Data were presented as the mean ± SEM, and a *p* value of < 0.05 was considered statistically significant. Schematic diagrams were generated using BioRender (https://www.biorender.com).

## Results

### Cholestyramine diet effectively sequesters the enterohepatic recirculation of bile acids *in vivo*

**Fig 1:**
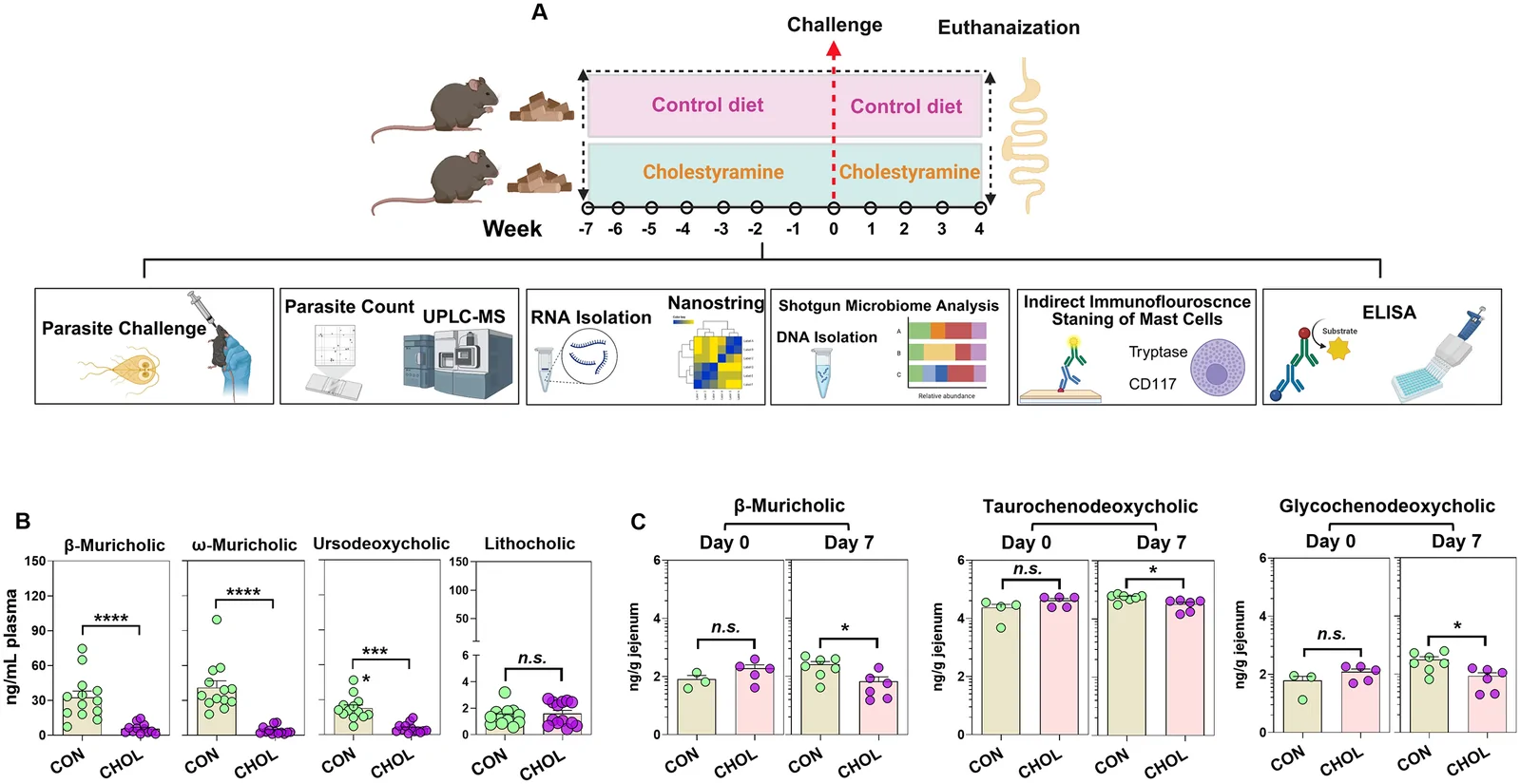
Cholestyramine diet effectively sequesters bile acids and prevents their reabsorption via enterohepatic circulation *in vivo.* **(A)** Experimental schematic design. Created in BioRender. Solaymani, **S.** (2026) https://BioRender.com/z54e5xy. **(B)** Age- and sex-matched 3-week-old female mice on a C57BL/6 background were given a control diet (*n* = 3-13) or a diet containing 2% cholestyramine (*n* = 3-13) for 7 weeks, and the plasma and (**C**) jejunal concentrations of representative primary (β-muricholic acid, tauro-chenodeoxycholic acid, glycochenodeoxycholic acid) and secondary bile acids (ω-muricholic acid, ursodeoxycholic acid, lithocholic acid) were assessed by targeted UPLC-MS/MS. The bile acid concentrations in plasma (ng/mL) and jejunum (ng/g tissue) were expressed as mean ± SEM. Uninfected control mice were maintained on either a control or cholestyramine diet for 7 weeks, as previously described, without exposure to *Giardia* infection. Data are pooled from two independent experiments. *p < 0.05; ****p < 0.0001, as determined by Mann-Whitney *U* test. *n.s*., not significant.

We fed mice a diet containing 2% cholestyramine *ad libitum* to sequester bile acids in the intestinal tract. Mice were randomized into two groups and given either a control (NIH-31 without cholestyramine) or a cholestyramine diet (NIH-31 containing 2% cholestyramine), starting 7 weeks before infection and continuing during *G. muris* infection (Fig 1A). A targeted UPLC-MS/MS analysis of plasma samples was performed to verify the efficient sequestration of bile acids after 7 weeks of feeding with a cholestyramine diet versus control-fed mice (Fig 1B). We confirmed the efficient sequestration of both primary and secondary bile acids in the plasma of mice receiving a cholestyramine diet. Furthermore, the concentrations of major bile acids in mice (β-muricholic acid, taurodeoxycholic acid, glycochenodeoxycholic acid) were significantly reduced in the jejunum of *G. muris*-infected mice receiving a cholestyramine diet compared with those fed a control diet (Fig 1C). However, the jejunal concentrations of other primary (taurocholic acid, glycocholic acid, cholic acid, α-muricholic acid, chenodeoxycholic acid) and secondary (lithocholic acid) bile acids were undetectable or remained unchanged 7 days after *G. muris* infection (S1 Fig).

### Bile acid sequestration increases resistance during the early phase of *G. muris* infection

**Fig 2:**
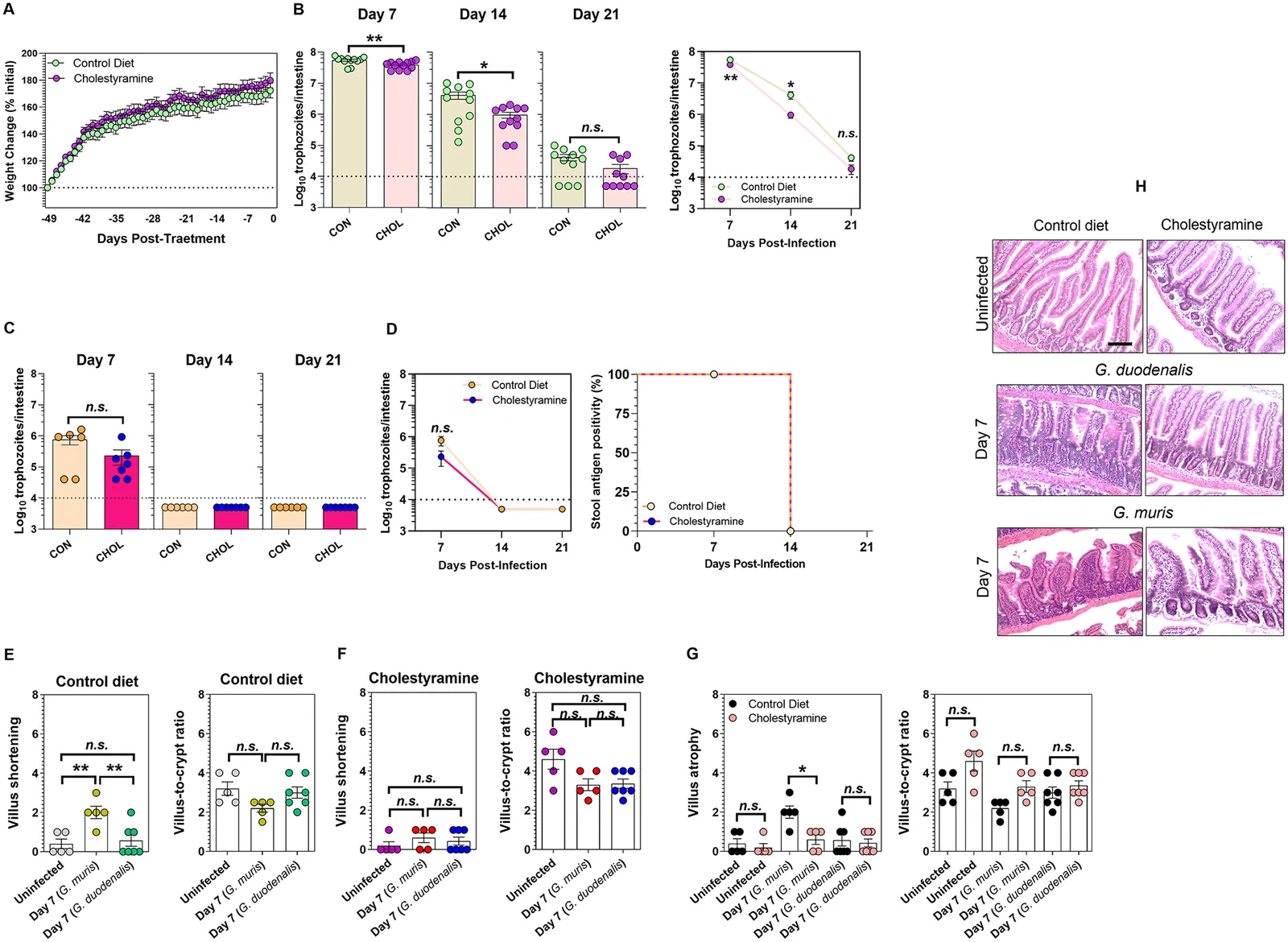
Bile acid sequestration renders mice more resistant to infection during the early phase of *G. muris* infection. **(A)** Body weight changes in age- and sex-matched C57BL/6 mice maintained on a control or cholestyramine diet for 7 weeks (*n* = 10/group). **(B)** Mice were infected with *G. muris* (10^4^ cysts/mouse) and **(C)** *G. duodenalis* (10^5^ cysts/mouse) via oral gavage. The numbers of live trophozoites at indicated time points were quantified using a hemocytometer, and expressed as trophozoites/intestine (mean ± SEM). The dashed lines represent the sensitivity of the conventional microscopy to detect *Giardia* trophozoites using a hemocytometer (10^4^ trophozoites/mL). *n* = 11-12/group **(B)**, and *n* = 6-7/group **(C)**. Data are pooled from two independent experiments. **(D)** The presence of *Giardia* antigens in the feces of infected mice was assessed during the course of *G. duodenalis* infection (*n* = 6-7/group). **(E)** Histological analysis of uninfected and *Giardia*-infected mice receiving either a control or cholestyramine (**F**) diet. **(G)** Pairwise comparisons of villus shortening scores and villus-to-crypt ratios in uninfected and *Giardia*-infected mice receiving either a control or cholestyramine (*n* = 5-7) diet. *p < 0.05; **p < 0.01, as determined by Mann-Whitney *U* test (**A-D**) or one-way ANOVA, followed by Tukey’s post-hoc adjustment for multiple comparisons. *n.s*., not significant. **(H)** Representative images of gross pathology in the mid-jejunum of C57BL/6 mice maintained on either control diet or a cholestyramine diet for 7 weeks (uninfected), and from mice examined 7 days after *G. muris* or *G. duodenalis* infection (*n* = 5-7). Villi in *G. muris*-infected mice receiving the control diet are noticeably shorter and blunted compared with uninfected controls (**H**, lower left). Magnification, × 20. Scale bar, 100 μm.

We next examined the potential effects of bile acid sequestration on the ability of host-specific (*G. muris*) and nonhost-specific (*G. duodenalis*) *Giardia* species to colonize C57BL/6 mice *in vivo*. Body weight changes in age- and sex-matched C57BL/6 mice maintained on either a control or cholestyramine diet for 7 weeks were not significantly different, and this remained unchanged 1 week after infection (Fig 2A and S2 Fig). These observations indicated that cholestyramine supplementation was not associated with taste aversion or reduced water and food intake, as body weights remained comparable between groups.

We found that *G. muris* trophozoites heavily colonized the small intestine of C57BL/6 mice fed a control diet, and these mice were eventually able to eradicate infection by day 21 (Fig 2B). However, mice receiving a cholestyramine diet showed significantly lower trophozoite numbers in the intestine at the peak of infection at day 7 (**p = 0.0022) and 14 (*p = 0.041) following *G. muris* infection (Fig 2B). Despite having significantly lower trophozoite numbers in the early phase of *G. muris* infection, C57BL/6 mice fed a cholestyramine diet were eventually able to clear *G. muris* infection, similar to those receiving the control diet. *G. duodenalis* infection resulted in transient colonization of the small intestine in mice fed either a control or cholestyramine diet (Fig 2C). Both cohorts were able to effectively control and eventually eradicate *G. duodenalis* infection, starting 14 days after infection (Fig 2C). A sensitive monoclonal antibody-based immunodetection assay of the feces [39,40] confirmed the efficient clearance of the *G. duodenalis* parasite (Fig 2D).

Collectively, these findings suggest that bile acid sequestration does not significantly impair adaptive immune-mediated parasite clearance during *Giardia* infection. Rather, the altered parasite burden during the early phase of infection likely reflects regulation of innate immune-dependent pathways.

Histological examination of H&E-stained jejunal tissue sections demonstrated significantly increased villus shortening in *G. muris*-infected mice receiving the control diet compared with uninfected controls, whereas *G. duodenalis* infection did not significantly increase villus shortening (Fig 2E). Importantly, treatment with cholestyramine for 7 weeks in the absence of infection did not alter jejunal architecture (Fig 2F), as evidenced by comparable villus shortening scores and villus-to-crypt ratios between cholestyramine-treated and control mice (Fig 2G). Moreover, cholestyramine-fed mice exhibited markedly reduced villus shortening during *Giardia* infection, indicating that bile acid sequestration attenuates infection-associated mucosal pathology (Fig 2F, and 2G). In contrast, neither *G. muris* nor *G. duodenalis* infection significantly altered villus-to-crypt ratios in mice fed either the control or cholestyramine diet (Fig 2E–2H).

### *Giardia* infection is characterized by intestinal mucosal mast cell activation and secretion of proinflammatory lipid mediators

**Fig 3:**
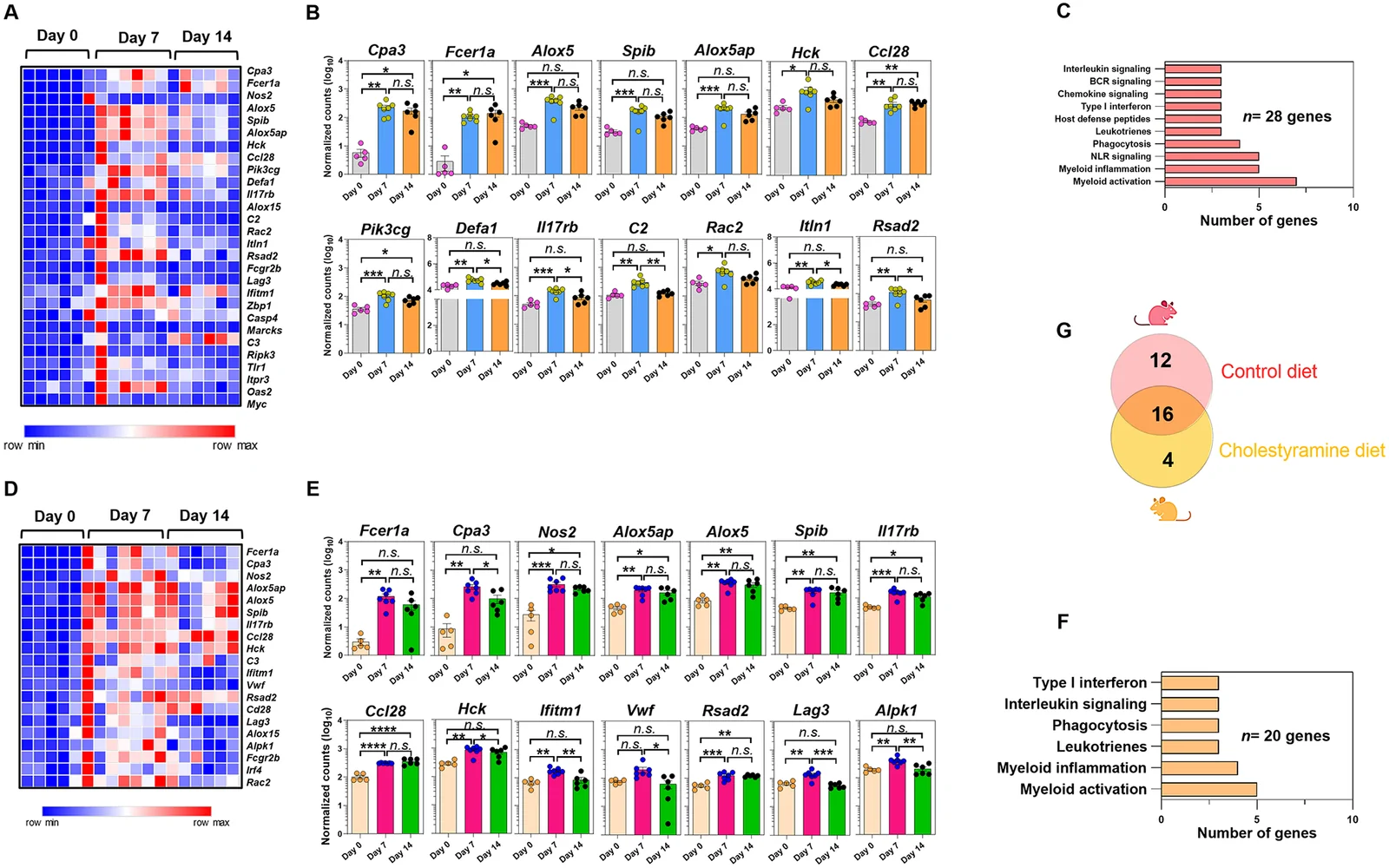
Increased mucosal mast cell activation signature is associated with *Giardia* infection. **(A,D)** Heatmaps of normalized expression of upregulated genes and **(B,E)**. top 14 highly upregulated transcripts in the jejunum of uninfected and *G. muris*-infected C57BL/6 mice fed a control (**A**,**B**) or cholestyramine (**D**,**E**) diet 7 and 14 days after infection, as determined by NanoString. In these experiments, total RNA was isolated from the mid-jejunum of uninfected (*n* = 5), *G. muris*-infected mice 7 (*n* = 7), and 14 (*n* = 6) days after infection, and the gene expression was analyzed by NanoString. (**C**,**F**) gene ontology analysis of biological processes enriched in the jejunum of *G. muris*-infected C57BL/6 mice 7 days after infection in mice fed a control diet (**C**) or a diet containing 2% cholestyramine **(F)**. **(G)** Venn diagram denoting the numbers of distinctively expressed, and overlapping genes between C57BL/6 mice fed a control or cholestyramine diet 7 days after *G. muris* infection. Results are shown as mean ± SEM. *p < 0.05; **p < 0.01; ***p < 0.001; ****p < 0.0001, as determined by a one-way ANOVA, followed by Tukey’s post-hoc adjustment for multiple comparisons. *n.s*., not significant.

In an unbiased screen for genes that were upregulated 7 and 14 days after *G. muris* infection, we discovered that a total of 28 genes (*n* = 28) were upregulated at least 2-fold in the jejunum of C57BL/6 mice fed a control diet compared with uninfected littermates (Fig 3A). The upregulated genes in the jejunum of *G. muris*-infected C57BL/6 mice receiving a control diet were mainly associated with mast cell activation, such as carboxypeptidase A3 (*Cpa3*) and the high-affinity IgE receptor (*Fcer1a*) (Fig 3A and 3B). Interestingly, transcripts for other classes of Fc receptors, such as those binding to IgG with low (*Fcgr2b, Fcgr4, Fcgrt*) and high (*Fcgr1*) affinity remained unchanged (S3 Fig). The gene ontology and biological pathway analysis revealed that the significantly upregulated genes were mainly enriched in 10 biological pathways (Fig 3C), with the majority involved in myeloid cell activation (*n* = 7), followed by myeloid cell inflammation (*n* = 5), NLR signaling (*n* = 5), and phagocytosis (*n* = 4). Transcripts responsible for the biosynthesis of leukotrienes from arachidonic acid (AA), such as arachidonate 5-lipoxygenase (*Alox5*; ***p = 0.0005) and arachidonate 5-lipoxygenase activating protein (*Alox5ap;* ***p = 0.0007), were significantly upregulated 7 days following infection relative to uninfected controls (Fig 3A–3C). However, genes participating in the biosynthesis of prostaglandins from AA, such as prostaglandin E receptors (*Ptger2, Ptger4*) and cyclooxygenase-2 (*Ptgs2*; Cox2), remained unchanged after *G. muris* infection (S4 Fig).

In mice fed a bile acid sequestration diet, we identified that a total of 20 genes (*n* = 20) were significantly upregulated 7 and 14 days after *G. muris* infection as compared with uninfected controls (Fig 3D). Genes associated with mast cell activation, such as *Fcer1a* and *Cpa3*, followed by those responsible for the biogenesis of leukotrienes were consistently the most highly expressed genes in the jejunum of *G. muris*-infected mice 7 and 14 days after infection during bile acid sequestration (Fig 3E). Upregulated genes were primarily enriched in six (*n* = 6) major biological processes, with myeloid cell activation (*n* = 5) being the most enriched, followed by myeloid cell inflammation (*n* = 4), leukotrienes (*n* = 3), and phagocytosis (*n* = 3) (Fig 3F).

As depicted in Fig 3G, 12/28 (42.85%) genes were uniquely upregulated in the jejunum of C57BL/6 mice fed a control diet, whereas 4/20 (20%) genes were only upregulated in mice fed a cholestyramine diet after *G. muris* infection. We also found that 16 genes were shared by the two cohorts after *G. muris* infection. We observed that the expression of genes responsible for the regulation of pyroptosis (*Casp4*) and necroptosis (*Zbp1*, *Ripk3*) were significantly upregulated in the jejunum of C57BL/6 mice 7 days after *G. muris* infection, consistent with observations that *Giardia* infection induces epithelial cell death in the intestine [50–54]. Notably, *Casp4*, *Ripk3*, and *Zbp1* transcripts remained unchanged in mice fed a bile acid sequestration diet, indicating that bile acids likely promote proinflammatory mucosal immune responses during intestinal insults by regulating death pathways such as inflammasome-mediated pyroptosis and necroptosis [13].

### Lack of FXR, but not TGR5, signaling in myeloid cells renders mice more resistant to enteric infection

**Fig 4:**
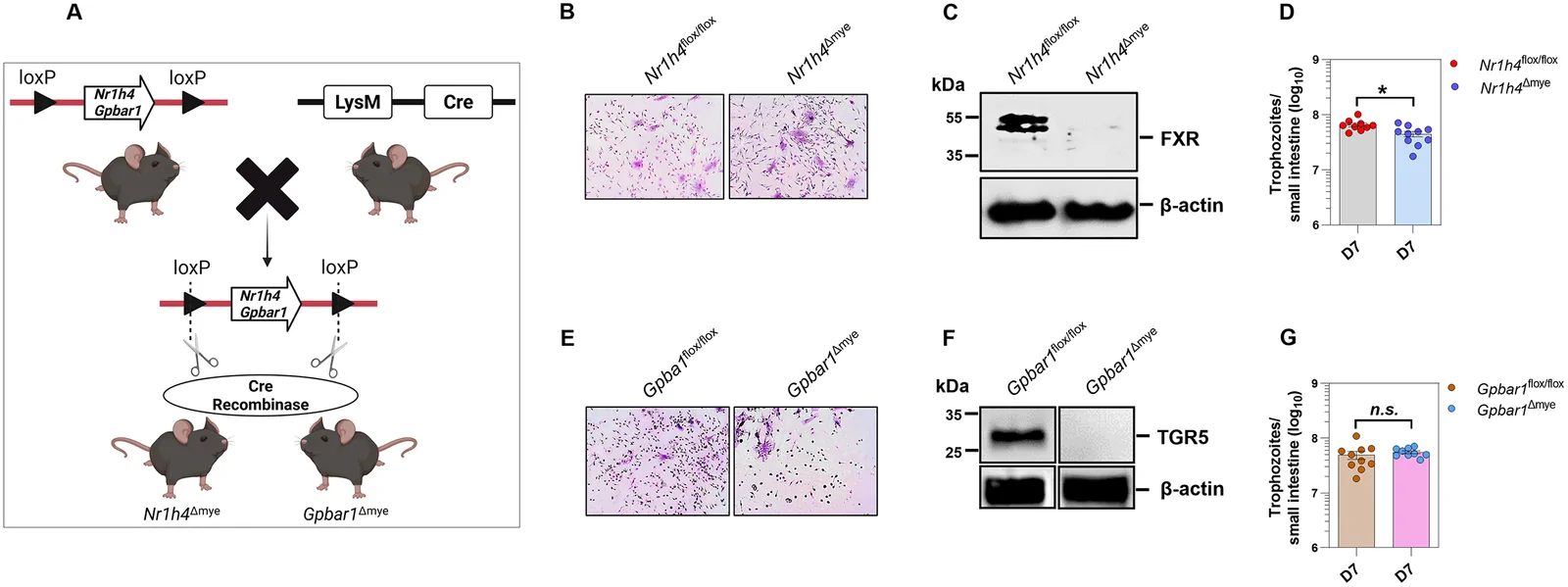
Mice with myeloid-specific deletion of FXR, but not TGR5, exhibited enhanced resistance to *Giardia* infection. **(A)** Breeding scheme to generate mice with myeloid-specific deletion of FXR and TGR5 using LysM-Cre-mediated recombination in *Nr1h4*^flox/flox^ and *Gpbar1*^flox/flox^ mice. Created in BioRender. Solaymani, **S.** (2026) https://BioRender.com/chaasz8. **(B)** Morphological characteristics of Giemsa-stained BMDMs from uninfected *Nr1h4*^flox/flox^ and *Nr1h4*^Δmye^ mice. **(C)** Western blot analysis validating the myeloid cell-specific deletion of FXR in BMDMs. **(D)** Trophozoite numbers in the entire small intestine of *Nr1h4*^Δmye^ mice (*n* = 10) and their *Nr1h4*^flox/flox^ littermates (*n* = 9) following *G. muris* infection. **(E)** Cellular characteristics of BMDMs from uninfected *Gpbar1*^flox/flox^ and *Gpbar1*^Δmye^ mice stained with Giemsa. **(F)** Efficient conditional deletion of TGR5 in differentiated BMDMs, as verified by Western blot analysis. **(G)** Trophozoite counts in the whole small intestine of *Gpbar1*^Δmye^ mice (*n* = 9) and their *Gpbar1*^flox/flox^ littermates (*n* = 10) following *G. muris* infection. Data are pooled from two independent experiments. *p < 0.05, as determined by Mann-Whitney *U* test. *n.s*., not significant.

Given the preponderance of myeloid cell activation- and inflammation-associated genes in the jejunum of *Giardia*-infected mice (Fig 3C and 3F), we investigated whether enhanced parasite clearance in cholestyramine-diet fed mice was mediated by bile acid receptors. Mice with conditional deletion of FXR (*Nr1h4*^Δmye^) or TGR5 (*Gpbar1*^Δmye^) in myeloid cells were generated (Fig 4A, 4B, and 4E), and the successful deletion of FXR and TGR5 in myeloid cells was validated by Western blot analysis (Fig 4C and 4F). Trophozoite numbers in the small intestine of *Nr1h4*^Δmye^ and *Gpbar1*^Δmye^ mice were counted after *G. muris* infection and compared with their littermates. We found that *Nr1h4*^Δmye^ mice exhibited accelerated parasite control (*p = 0.014) at day 7 post-infection compared with their *Nr1h4*^flox/flox^ littermates (Fig 4D), whereas *Gpbar1*^Δmye^ and *Gpbar1*^flox/flox^ mice had comparable trophozoite numbers in the small intestine at day 7 after infection (Fig 4G). These findings suggest that the lack of myeloid cell-specific FXR signaling enhances parasite control during *Giardia* infection.

### Mast cells are activated after *Giardia* infection

**Fig 5:**
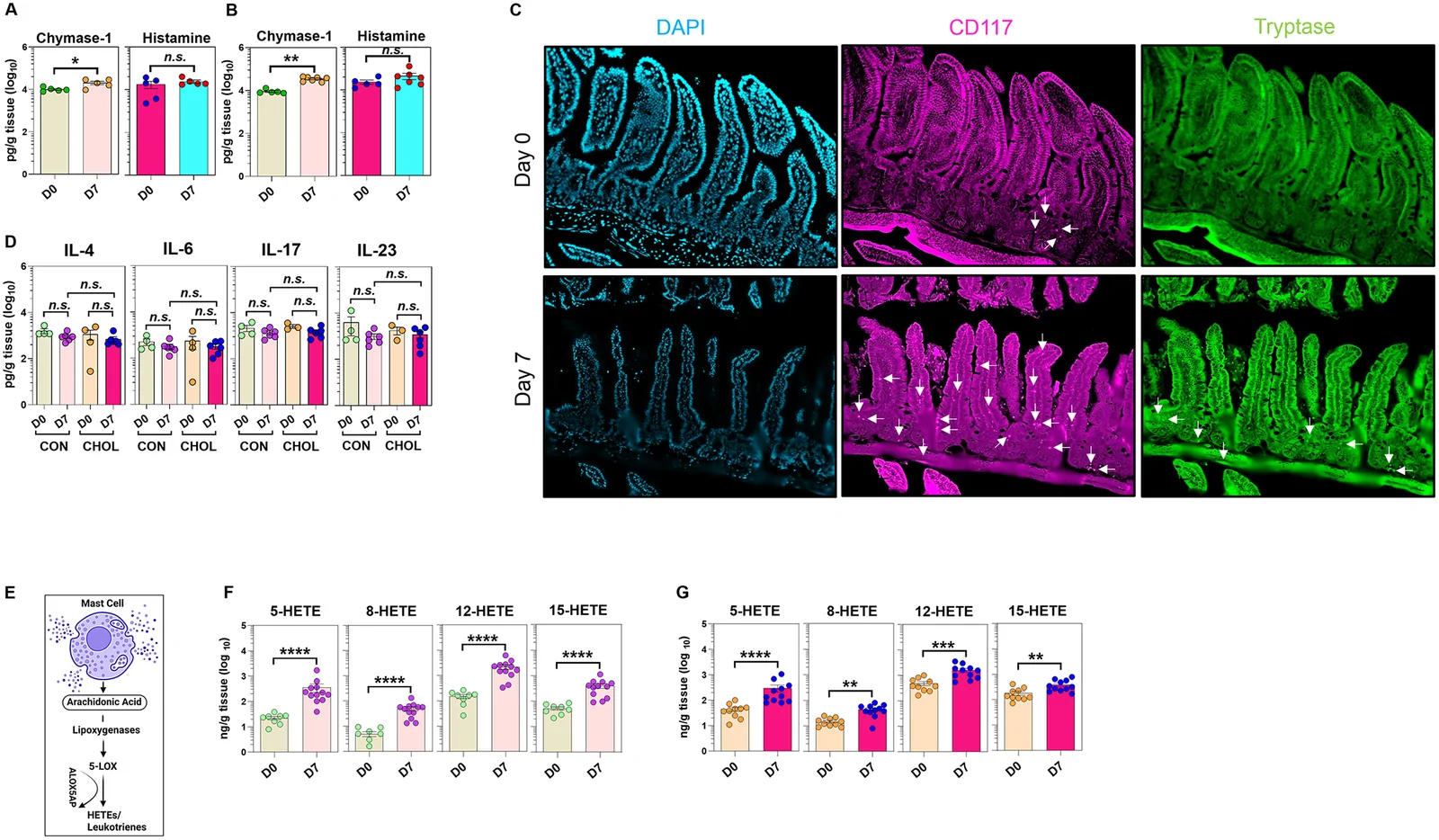
Mucosal mast cell activation during *Giardia* infection is accompanied by increased secretion of proinflammatory lipid mediators in the small intestine. **(A)** Release of mast cell effector protease chymase-1 and histamine in uninfected, *G. duodenalis-* and **(B)** *G. muris-*infected C57BL/6 mice, 7 days after infection, as assessed by ELISA. The entire mid-jejunum (10 cm) was cultured in RPMI-1640 for 24 h at 37 °C in a 5% CO2 incubator, and the protein concentrations of chymase-1 and histamine were assessed by ELISA. The concentrations of released proteins were normalized as pg/g tissue and expressed as mean ± SEM (*n* = 5-7/condition/time point). **(C)** Mast cell (CD117^+^ tryptase^+^) frequency in the jejunum of uninfected and *Giardia*-infected mice 7 days after infection (20 × magnification). **(D)** IL-4, IL-6, IL-17, and IL-23 release in the jejunum of *G. muris*-infected mice fed a control or cholestyramine diet, as measured by ELISA (*n* = 3-6/time point/diet). **(E)** Diagram illustrating the biosynthetic pathways of AA-derived proinflammatory lipid mediators, including HETEs. Created in BioRender. Solaymani, **S.** (2026) https://BioRender.com/rcsfjyi. **(F)** Jejunal concentrations of HETE stereoisomers 5-HETE, 8-HETE, 12-HETE, and 15-HETE were assessed by targeted UPLC-MS/MS. in mice fed a control or (**G**) cholestyramine diet before and 7 days after *G. muris* infection. Data were pooled from two repeat experiments using 8-10 mice/cohort (uninfected) and 11-12/cohort (infected). *p < 0.05, **p < 0.01, ***p < 0.001; ****p < 0.0001, as determined by Mann-Whitney *U* test or a one-way ANOVA, followed by Tukey’s post-hoc adjustment for multiple comparisons. *n.s*., not significant.

We further confirmed the activation of mucosal mast cells by measuring the release of chymase-1, also known as mast cell chymase, in the jejunal tissue explants of uninfected and *Giardia*-infected mice. Protein levels of chymase-1, but not histamine, were significantly increased in the jejunum of C57BL/6 mice infected with *G. muris* (*p = 0.031) or *G. duodenalis* (**p = 0.0025) 7 days after infection compared with uninfected controls (Fig 5A and 5B). Consistent with elevated secretion of chymase-1, we observed an increased frequency of mast cells, identified by dual CD117 and tryptase staining, in the jejunum of *Giardia*-infected mice (day 7) compared with uninfected mice (Fig 5C). We next evaluated how bile acid sequestration impacted T cell (IL-17) and non-T cell derived (IL-6, IL-23) cytokines. No significant increase in the secretion of IL-4, IL-6, IL-17, and IL-23 was observed in the jejunum of *G. muris*-infected mice irrespective of the diet (Fig 5D).

### Proinflammatory HETE biosynthesis is activated after *Giardia* infection

Mast cells are key regulators of proinflammatory immune responses, expressing a diverse array of AA-derived proinflammatory lipid mediators, such as prostaglandins and leukotrienes [55–57]. Despite the well-established roles of mast cells in protection against *Giardia* infection [38,58–61], the mechanisms by which proinflammatory immune responses at the mucosal surfaces of the small intestine are regulated by mast cells during *Giardia* infection in humans and in murine hosts are not fully understood. We discovered robust intestinal mucosal mast cell activation and degranulation after *Giardia* infection which was associated with elevated expression levels of a wide array of AA-derived proinflammatory lipid mediators in the jejunum of *G. muris*-infected C57BL/6 mice fed either a control or cholestyramine diet (Fig 5E–5G).

Targeted UPLC-MS/MS metabolomics revealed significant increases in jejunal tissue concentrations of HETE stereoisomers 5-HETE (~16-fold; ****p < 0.0001), 8-HETE (~10-fold; ****p < 0.0001), 12-HETE (~14.73-fold; ****p < 0.0001), and 15-HETE (~7.7-fold; ****p < 0.0001) at day 7 after infection compared with uninfected mice (Fig 5F and 5G). Pairwise comparisons revealed no significant differences in the jejunal concentrations of HETE stereoisomers between mice fed a control diet and those fed cholestyramine, regardless of diet or infection (Fig 5F and 5G). These observations highlight the importance of the leukotriene pathway, rather than the canonical COX-1/2-prostaglandin axis, in the development and maintenance of immunopathological sequelae in giardiasis.

### Bile acid sequestration enhances the richness and diversity of microbial composition

**Fig 6:**
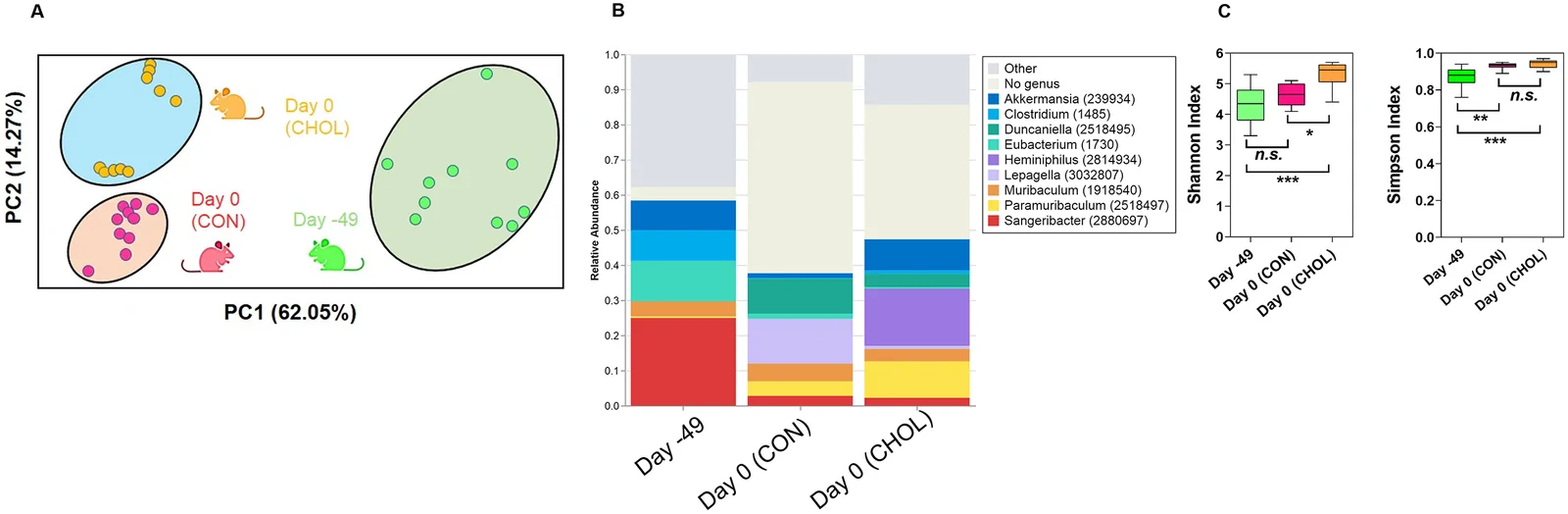
Bile acid sequestration significantly enhances the microbiome diversity and richness. **(A)** Principal component analysis of the beta diversity, (**B**) relative abundance, and (**C**) the alpha diversity of fecal microbiome in mice before (*n* = 10/cohort) and after (*n* = 10) bile acid sequestration, as compared with mice fed a control diet (*n* = 10) in the absence of infection. Age- and sex-matched C57BL/6 mice fed a control or cholestyramine diet for 7 weeks, and the fecal microbiome was analyzed using a shotgun metagenomic approach. Values are mean ± SEM. *p < 0.05; **p < 0.01; ***p < 0.001, as determined by a one-way ANOVA, followed by Tukey’s post-hoc adjustment for multiple comparisons. *n.s*., not significant.

We used a shotgun metagenomic analysis of the fecal microbiome to better understand the potential impact of bile acid sequestration on the diversity and composition of the microbiome. The PCA analysis of beta diversity indicated distinct gut microbiome signatures at the genus level (PC1, 62.05%; PC2, 14.27%) prior to bile acid sequestration and after 7 weeks of feeding with either a control or cholestyramine diet (Fig 6A). At day –49, the most abundant bacteria in the feces of mice were *Sangeribacter* (*S. muris*; 25.00 ± 2.43%), *Akkermansia* (*A. muciniphila*; 15.49 ± 3.38%), and *Lactobacillus* (*L. johnsonii*; 11.58 ± 1.49%) (Fig 6B). Relative abundance of bacteria in the genera *Lactobacillus* (0.82 ± 0.08%), *Akkermansia* (3.36 ± 1.17%) and *Sangeribacter* (1.87 ± 0.097%) significantly decreased, while *Heminiphilus* (*H. faecis*; 10.15 ± 1.88%), *Paramuribaculum (P. intestinale*; 7.27 ± 0.48%), *Duncaniella* (*D. dubosii*; 5.70 ± 0.828%), *Bacteroides* (*Bacteroides* spp.; 3.73 *±* 0.27%), and *Prevotella* (*Prevotella* spp.; 3.13 ± 0.307%) were the most abundant bacterial genera in the feces 7 weeks after receiving the cholestyramine diet (Fig 6B).

Bile acid sequestration expanded fecal microbial richness and diversity, as evidenced by significantly higher Shannon indices before treatment (4.33 ± 0.189) and after feeding with a control (4.66 ± 0.109) or cholestyramine (5.29 ± 0.142) diet (Fig 6C). The Simpson index increased significantly both before and after bile acid sequestration, whereas no statistical differences were observed in this index between mice fed a control (0.93 ± 0.006) or cholestyramine (0.943 ± 0.007) diets for 7 weeks (Fig 6C).

**Fig 7:**
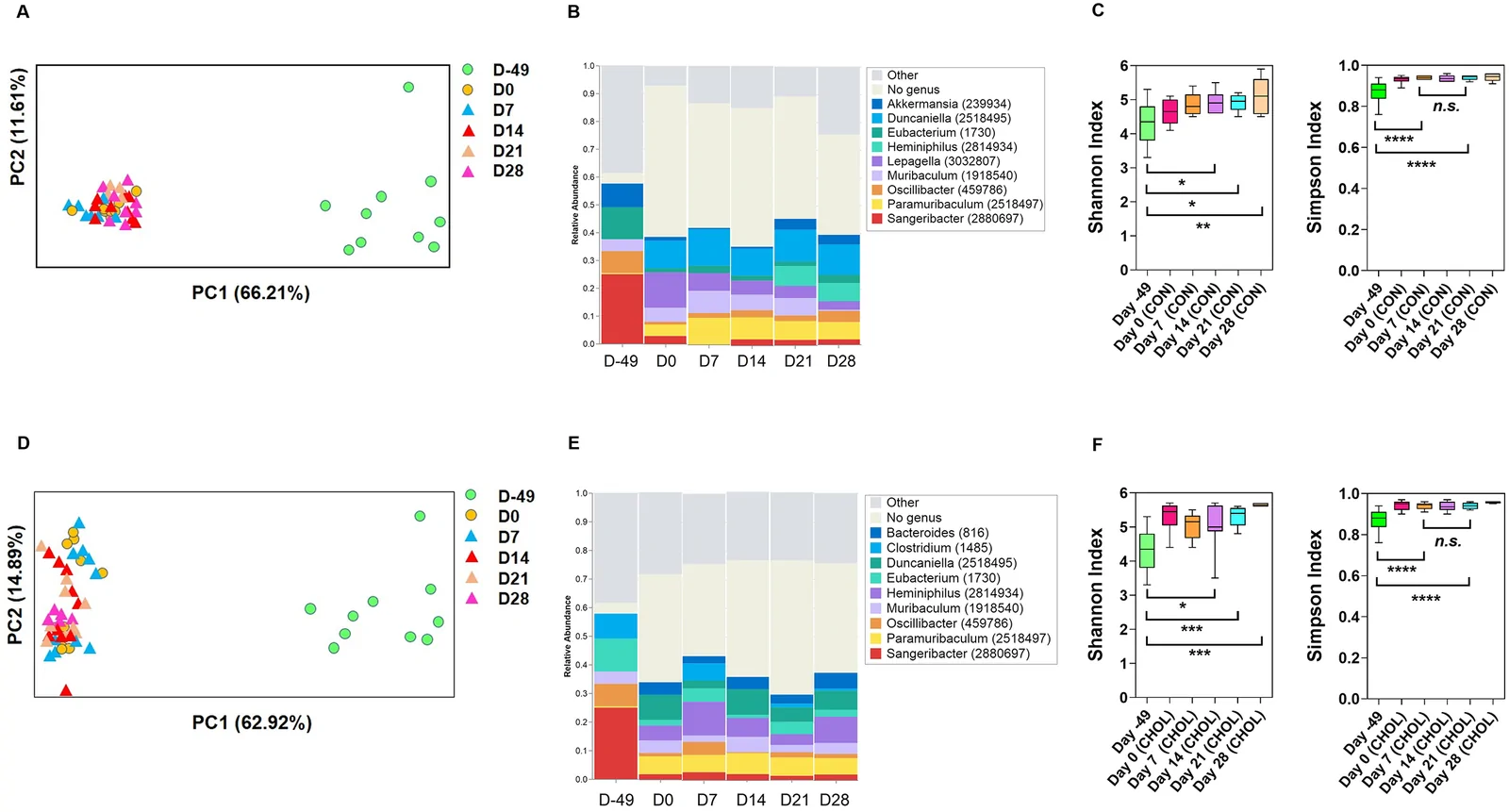
Fecal microbiome diversity signature during *Giardia* infection. **(A)** Principal component analysis of the beta diversity, (**B**) relative abundance, and (**C**) the alpha diversity of the fecal microbiome in mice fed a control or cholestyramine (**D-F**) diet over time before (day –49) and after bile acid sequestration as well as during the entire course of *G. muris* infection (days 7, 14, 21, 28). Mice were given a control or cholestyramine diet for 7 weeks, and were infected with *G. muris*. Fecal pellets were aseptically collected, and the fecal microbiome was assessed using a shotgun metagenomic approach (*n* = 4-10 cohort/time point). Data were expressed as mean ± SEM **(C,F)**. *p < 0.05; **p < 0.01; ***p < 0.001, as determined by a one-way ANOVA, followed by Tukey’s post-hoc adjustment for multiple comparisons. *n.s*., not significant.

Next, we investigated *G. muris*-specific effects on the microbial diversity and richness of the fecal microbiome over time in mice fed a control diet, independent of bile acid sequestration. Bacteria in the genera *Duncaniella* (12.93 ± 0.939%), *Paramuribaculum (P. intestinale*; 9.41 ± 0.385%), *Lepagella* (*L. muris*; 5.79 ± 0.507%), and *Muribaculum* (4.11 ± 1.028%) were the most abundant genera identified in *G. muris*-infected mice 7 days after infection (Fig 7A and 7B). Notably, the abundances of bacteria in the genera *Paramuribaculum*, *Muribaculum*, and *Duncaniella* were increased, whereas *Lepagella* spp. abundance decreased 7 days after *G. muris* infection. However, the relative abundance of *Eubacterium* (1.78 ± 0.264%) and *Sangeribacter* (1.32 ± 0.446%) at day 7 after infection remained unchanged compared with uninfected mice, although significantly lower than those of day –49. We investigated the fecal microbiome 14 and 21 days after *G. muris* infection, the time points at which C57BL/6 mice typically mount effective *Giardia*-specific adaptive immunity to eradicate infection [35,36]. The dominant bacterial genera 14 and 21 days after *G. muris* infection were *Duncaniella* spp., *Lepagella* spp., and *Muribaculum* spp. (Fig 7B). Shannon indices were significantly higher at 14 (4.93 ± 0.101) and 21 (4.93 ± 0.075), and 28 (5.11 ± 0.24) days after *G. muris* infection compared with day –49 but not days 0 and 7 after infection (Fig 7C).

We next investigated the potential impact of infection on the fecal microbial profile upon the resolution of *G. muris* infection. *Duncaniella* spp. (10.03 ± 1.00%), *Heminiphilus* spp. (7.12 ± 1.56%), *Alistipes* spp. (5.48 ± 1.167%), and *Akkermansia* spp. (4.4 ± 1.137%) were the most abundant bacteria discovered in the feces at the clearance phase of *G. muris* infection (Fig 7B). Notably, we found significantly higher microbial richness and diversity in mice that cleared *G. muris* infection 28 days after infection, as indicated by significantly higher Shannon indices (5.11 ± 0.24%) than those at day –49 (Fig 7C). These findings underscore the persistent microbial dysbiosis following *G. muris* infection, even after the infection is efficiently eradicated.

The microbial signature in an experimental *G. muris* infection model with bile acid sequestration was investigated. *Heminiphilus* (9.96 ± 2.07%) was the most abundant bacterial genus in bile acid-sequestered mice 7 days after infection, followed by *Paramuribaculum* spp. (7.17 *±* 0.429), *Duncaniella* spp. (6.90 ± 1.491%), *Akkermansia* spp. (3.56 ± 1.006), *Bacteroides* spp. (3.41 ± 0.6%), *Muribaculum* spp. (2.94 ± 0.401%), and *Sangeribacter* spp. (2.85 ± 0.245%) (Fig 7D and 7E). Alpha diversity, as evidenced by the Shannon indices, was also significantly higher than that of mice at day –49 (Fig 7F).

**Fig 8:**
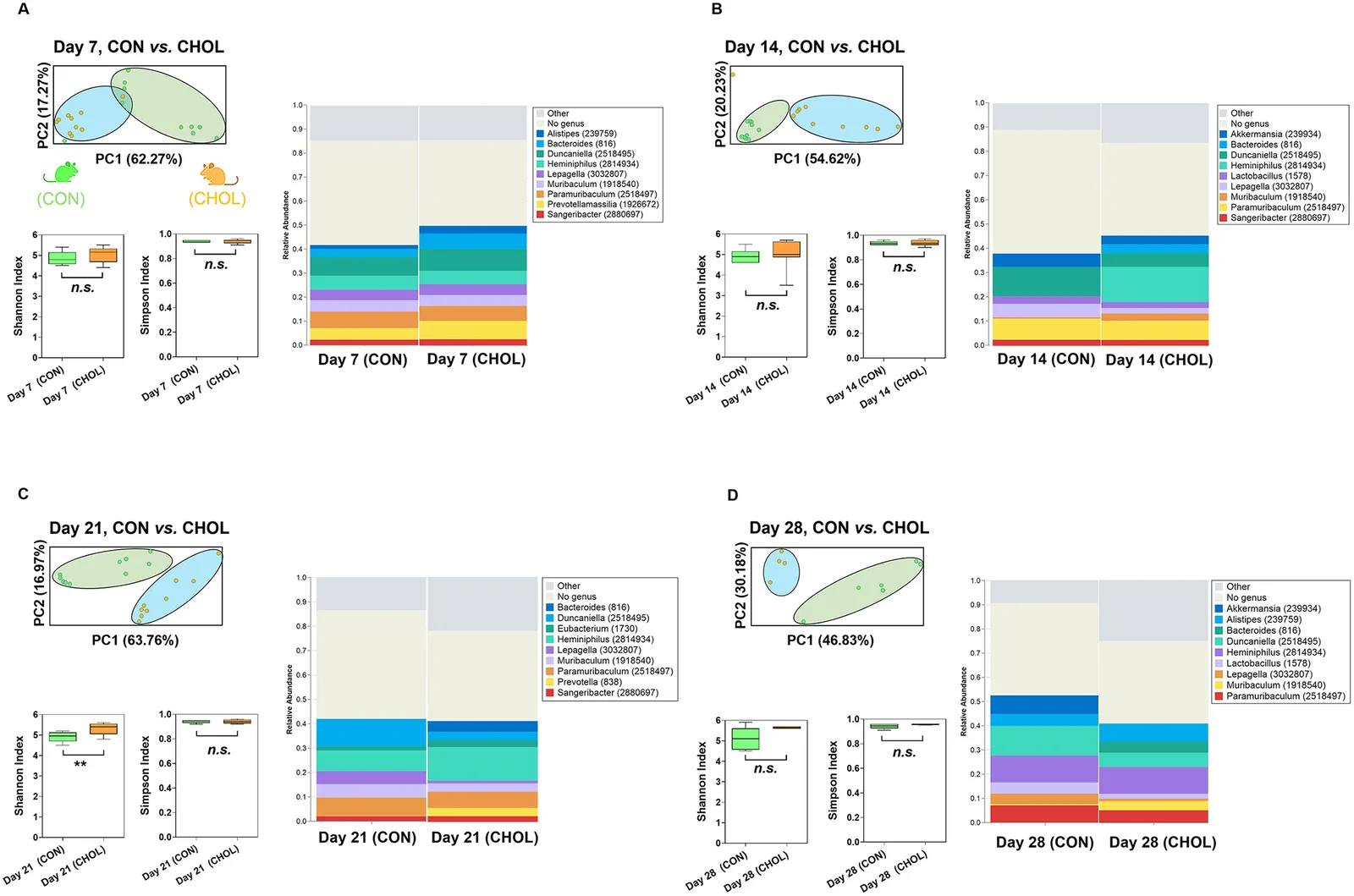
Comparison of the fecal microbiome between mice fed a control or cholestyramine diet during the entire course of *Giardia* infection. (**A**) pairwise comparisons of the fecal microbiome beta diversity, relative abundance, and alpha diversity between mice fed a control or cholestyramine diet over time at day 7, (**B**) day 14, (**C**) day 21, and (**D**) day 28 following *G. muris* infection. Mice were given a control or cholestyramine diet for 7 weeks, and were infected with *G. muris*. Fecal pellets were aseptically collected, and the fecal microbiome was assessed using a shotgun metagenomic approach (*n* = 4-10 cohort/time point). Data were expressed as mean ± SEM. *p < 0.05; **p < 0.01; ***p < 0.001, as determined by Mann-Whitney *U* test. *n.s*., not significant.

Further pairwise PCA comparisons indicated distinct gut microbiome clusters in infected mice in the absence or presence of bile acids 7, 14, and 21 days after *G. muris* infection (Fig 8A–8D). The relative abundance of *Heminiphilus* spp. expanded in bile acid-sequestered mice 7 (9.96 ± 2.073%) and 14 (9.04 ± 1.840%), and 21 (8.99 ± 1.746%) days after infection (Fig 8A–8C). Diversity and richness of the two cohorts remained unchanged at days 7 and 14, but increased significantly at day 21 after *G. muris* infection (Fig 8A–8C). The relative abundance of *Duncaniella* spp. and *Heminiphilus* spp., *Alistipes* spp*.* and *Paramuribaculum* spp. were comparable during the clearance phase of *G. muris* infection regardless of the diet, whereas *Akkermansia* spp. were almost exclusively detected in *G. muris*-infected mice fed the control diet (Fig 8D).

The lack of significant differences in overall microbiome composition at days 7 and 14, despite reduced parasite burden, suggests that early protection may be mediated by functional changes or specific microbial taxa not identified by global community analyses. By day 21, persistent microbiome differences despite comparable parasite burdens indicate that microbiome remodeling primarily influences the early establishment of infection.

### Antibiotic-driven depletion of gut microbiome renders mice more susceptible to *Giardia* infection

**Fig 9:**
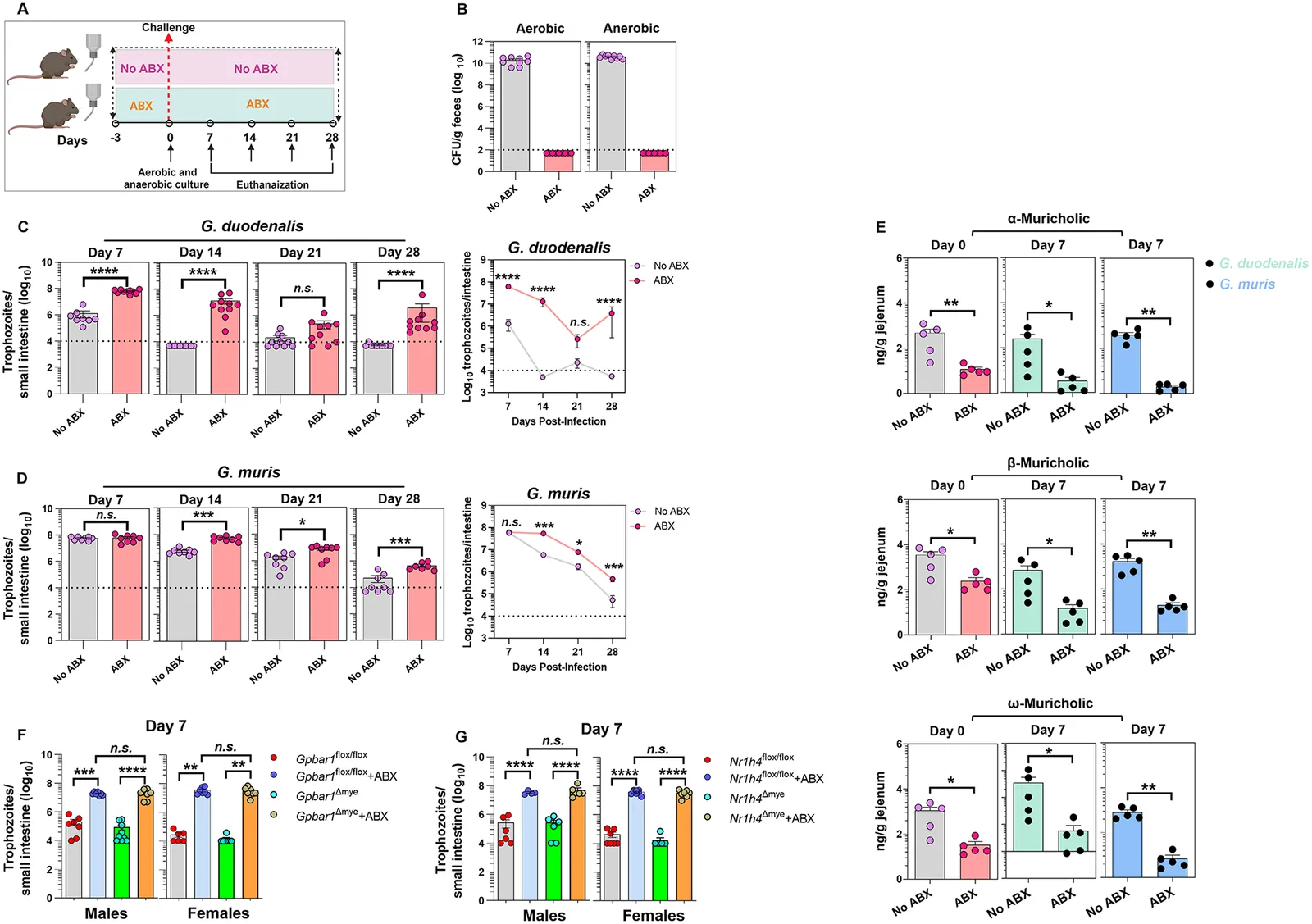
Antibiotic-driven depletion of gut microbiome renders mice susceptible to *Giardia* infection. **(A)** Schematic representation of study design. Created in BioRender. Solaymani, **S.** (2026) https://BioRender.com/t6zzasi. **(B)** Efficient depletion of aerobic and anaerobic commensal bacteria following the treatment of C57BL/6 mice with an antibiotic cocktail containing ampicillin, neomycin, and vancomycin for 3 days prior to infection. **(C)** *G. duodenalis* and **(D)** *G. muris* infection kinetics were evaluated in antibiotic-treated mice relative to untreated mice (no antibiotics). **(E)** Tissue concentrations of the primary (α- and β-muricholic) and secondary (ω-muricholic) bile acids measured in the jejunum of uninfected and infected C57BL/6 mice following infection with *G. duodenalis* and *G. muris*, with or without antibiotic treatment. **(F)** *G. duodenalis* infection in *Gpbar1*^Δmye^ and **(G)** *Nr1h4*^Δmye^ mice and their littermate controls with or without antibiotic treatment. The dashed lines indicate the detection limit of conventional microscopy for identifying *Giardia* trophozoites with a hemocytometer (10⁴ trophozoites/mL). Data were expressed as mean ± SEM of *n* = 4-10 per time point. Data are pooled from two independent experiments (**B-D,F,G**). Data are representative of two independent experiments with similar results **(E)**. *p < 0.05, **p < 0.01, ***p < 0.001, ****p < 0.0001, as determined by Mann-Whitney *U* test (**C,D,E**) and one-way ANOVA **(F,G)**, followed by Tukey’s post-hoc adjustment for multiple comparisons. *n.s*., not significant.

We have previously demonstrated that mice pretreated with broad-spectrum antibiotics exhibited increased susceptibility to *G. duodenalis* infection [35,36]. We next examined the extent to which perturbations of commensal bacterial communities in the intestinal tract impacted the course of *Giardia* infection (Fig 9A).

We observed efficient depletion of aerobic and anaerobic bacteria in C57BL/6 mice pretreated with an antibiotic cocktail for 3 days compared with control mice receiving no antibiotics (Fig 9B). These findings confirm that antibiotic intake was sufficient and comparable across treated animals. Notably, mice pretreated with an antibiotic cocktail prior to infection exhibited significantly greater numbers of *G. duodenalis* (Fig 9C) and *G. muris* (Fig 9D) trophozoites/small intestine relative to age- and sex-matched C57BL/6 control mice with no prior antibiotic treatment.

We next assessed the impact of antibiotic treatment on representative major murine primary (α- and β-muricholic acids) and secondary (ω-muricholic acid) bile acid pools in uninfected and *Giardia*-infected C57BL/6 mice in the absence or presence of antibiotic treatment. We found that antibiotic pretreatment significantly reduced α-, β-, and ω-muricholic acid levels prior to infection, as evidenced by the differences between antibiotic-treated and untreated mice prior to infection (Fig 9E, left panels). Notably, jejunal primary and secondary bile acid levels remained relatively unchanged between uninfected and *Giardia*-infected mice that did not receive antibiotics (Fig 9E). The antibiotic-induced differences in the bile acid pool became even more pronounced in *G. muris*-infected mice, but not in those infected with *G. duodenalis*, by day 7 post-infection (Fig 9E). Collectively, these findings demonstrate that antibiotic-driven microbiome disruption reshapes the jejunal bile acid microenvironment even before infection and that these alterations are further amplified during *Giardia* infection.

Finally, we investigated the extent to which host bile acid receptor signaling and gut microbiome-mediated mechanisms contributed to the regulation of mucosal defense against *Giardia* infection *in vivo*. To this end, we simultaneously combined specific deletion of TGR5 and FXR in myeloid cells and gut microbiome disruption driven by antibiotics. We observed that disruption of gut microbiome profoundly determined parasite control in the intestine, as the oral administration of a broad-spectrum antibiotic cocktail resulted in significantly higher trophozoite numbers (~100–1000-fold) in the small intestine of both genotypes/sexes, as compared with their littermate controls receiving no antibiotics (Fig 9F and 9G). These findings indicated that the protective effects of diet-induced bile acid sequestration or FXR deficiency in myeloid cells were abolished upon microbiome depletion. These observations highlighted the significance of microbiome-dependent mechanisms, rather than bile acid receptor signaling pathways alone, as a critical determinant of *Giardia* parasite control.

## Discussion

We observed that mice fed a diet containing a bile acid sequestrant, cholestyramine, had significantly lower trophozoite numbers early in *G. muris* infection. Enhanced resistance to *Giardia* infection was dependent on FXR, but not TGR5, signaling in myeloid cells. However, the ability of mice to eradicate parasites was not compromised during bile acid sequestration in mice fed a cholestyramine diet as compared with those fed the control diet. Significant activation of intestinal mucosal mast cells and the expression of proinflammatory lipid mediators were associated with *Giardia* infection in the jejunum. We also found that bile acid sequestration did not substantially change the immune signature in *G. muris*-infected intestinal tissue, nor alter key AA-derived proinflammatory lipid mediators, such as HETEs. Bile acid sequestration, however, had a profound impact on the diversity and richness of bacterial communities in the intestine of mice fed a diet containing a bile acid sequestrant. Finally, we observed that the antibiotic-driven depletion of the gut microbiome led to significantly reduced jejunal primary and secondary bile acid pools and increased susceptibility to *Giardia* infection, indicating the requirement for intact gut microbial communities for host resistance to enteric infection.

Mast cells are enriched at intestinal mucosal surfaces and function as effectors that amplify T helper 2 (T_H2_)-mediated proinflammatory and allergic conditions [62,63]. Upon activation by T_H2_ cytokines, these cells release proinflammatory lipid mediators that can drive allergy-like manifestations reported in human giardiasis [62–65].

The findings of the current study are consistent with previous reports demonstrating mast cell activation after *Giardia* infection [38,58–61]. Adherence of *Giardia* trophozoites to the epithelial surfaces of the small intestine triggers a robust T_H2_-polarized immune activation, marked by an increase in the frequency of CD4^+^ T cells expressing the canonical T_H2_ transcription factor GATA3 [35,66,67]. *Giardia*-induced immune activation is accompanied by the release of prototypical T_H2_ cytokines, such as IL-4 and IL-13, and mast cell hyperplasia in the small intestine [35,68]. Live *G. duodenalis* parasites, as well as their excretory-secretory products can activate mast cells, leading to secretion of TNF-α and IL-6 [68,69]. Mast cells also participate in innate immune defense during *Giardia* infection, and mice lacking mast cells fail to control *Giardia* infection [70,71]. Mast cells enhance intestinal smooth muscle contractility and promote intestinal motility to expel parasites, and pre-treatment of mice with mast cell stabilizers, such as ketotifen, abolished the smooth muscle contractions in mice infected with *G. duodenalis* [60].

Mast cells have also been implicated in the development and exacerbation of allergic manifestations commonly observed in human giardiasis, suggesting a dual role for mast cells during *G. duodenalis* infection in humans [59,60]. Understanding the mechanisms underpinning mast cell-mediated regulation of protective and deleterious immune responses during giardiasis can provide insight into the development of novel preventive and therapeutic strategies to improve mucosal immune responses to enteric microbial infections.

Regulation of proinflammatory immune responses during enteric microbial infections is tightly regulated to limit persistent inflammation and tissue damage [45,72]. AA metabolism is a key step in the biosynthesis of a variety of proinflammatory lipid mediators, such as prostaglandins and leukotrienes [41,73–75]. Mice deficient for *Alox5* or immunocompetent mice receiving zileuton, a selective ALOX5 inhibitor, were more susceptible to infection with the intracellular protozoan parasite *Leishmania amazonensis* [76]. Aspirin, a cyclooxygenase pathway inhibitor, promoted leukotriene biosynthesis and further exacerbated cerebral malaria both in humans and in animal models of human malaria, indicating the dual roles of leukotrienes in the regulation of proinflammatory immune responses in various proinflammatory settings [77,78]. Although the roles of leukotrienes, as end-products of AA metabolism, during microbial infections are well established, it is still unclear how the activation of the leukotriene pathway in the absence of the prostaglandin pathway contributes to the development and exacerbation of clinical symptoms observed during human giardiasis. It is also unclear how targeting the regulation of AA metabolism via HETEs, which function further upstream of the leukotriene pathway, could modulate immune responses during giardiasis. Our findings suggest that a predominance of the leukotriene pathway in the absence of prostaglandin biosynthesis may provide novel insights into the regulation of proinflammatory and allergic responses during giardiasis.

Our study showed no significant increase in IL-4, IL-6, IL-17, or IL-23 secretion in whole jejunal explants from uninfected and *Giardia*-infected C57BL/6 mice, regardless of diet. Although our previous studies demonstrated a significant induction of IL-4 and IL-17 following *Giardia* infection, those analyses were performed in the spleen and gut-draining inductive sites (i.e., mesenteric lymph nodes; MLNs), where antigen-specific T cells are abundant [35,79]. In contrast, the present study examines the effector compartment, the whole small-intestinal lamina propria (LP), which represents a heterogeneous tissue dominated by intestinal epithelial cells (IECs), innate effector subsets, and relatively few antigen-specific T cells [80,81]. This compartmental difference likely explains why cytokine induction observed in the spleen and small-intestinal draining lymph nodes does not necessarily correlate with cytokines measured by explant-based intestinal cytokine assays.

Bile acid metabolism by commensal bacteria is critical for inducing peripheral tolerance in the colon [16,82]. Bile acids shape the gut microbiome directly through antimicrobial detergent activity or indirectly via signaling pathways downstream of their cognate receptors [83,84]. Bile acids and their microbially-transformed metabolites can promote the development of both innate and adaptive immunity, leading to enhanced protection against enteric microbial pathogens [85,86]. However, the gut microbiome dysbiosis in infectious and non-infectious intestinal disorders can reduce the luminal bile acid pool, resulting in impaired bile acid homeostasis [11,87]. These findings were further supported by the observations that experimental animals fed exogenous bile acids or their analogs were prone to significant changes in the microbial composition [88,89].

Despite these observations, how bile acid sequestration affects the luminal bile acid pool and the gut microbiome composition remains unknown. A prior study by Erlandsen [90] showed that a diet containing 5% cholestyramine reduced the excretion of *G. muris* cysts in the feces compared with the control diet, while trophozoite burdens remained numerically similar. This study, however, employed the cholestyramine diet only for short durations rather than the 7 weeks used in our study, and did not explore potential underlying immune- or nonimmune-mediated mechanisms driving this reduction.

Our findings that bile acids may function as limiting factors for the expansion of microbial ecosystems are particularly significant. The gut microbiome is essential for effective cholesterol metabolism, as certain bacterial species can metabolize and potentially lower cholesterol levels [91,92]. It remains unclear whether the cholesterol-lowering properties of bile acid sequestrants are mediated directly by binding to luminal bile acids or indirectly by selectively promoting the diversity and richness of cholesterol-metabolizing bacterial species.

The role of the gut microbiome in susceptibility to *G. duodenalis* was highlighted by the observations that isogenic mice obtained from different commercial sources exhibited variable susceptibility to *Giardia* infection, while resistance to *G. duodenalis* infection was transferable to susceptible mice when co-housed with mice that were innately resistant to infection [37]. Emerging evidence suggests that a diverse gut microbiome signature is positively associated with enhanced resistance to enteric microbial infection [33,93], and that host gut microbiome disparity may contribute to the development of clinical symptoms during infection with enteric microbial pathogens [42]. This is consistent with prior studies demonstrating that the antibiotic-driven depletion of the gut microbiome renders mice more susceptible to infection with *G. duodenalis* [35–37]. These findings also reinforce the significance of microbial diversity as a protective factor in host-pathogen interactions at the intestinal mucosal surfaces, whereas microbiome depletion may create a permissive microenvironmental niche that facilitates robust trophozoite colonization and persistence. However, it is important to distinguish the effects of bile acid sequestration from those caused by antibiotic treatment. Cholestyramine remodels microbial community composition, whereas broad-spectrum antibiotics generally deplete commensal bacteria. Accordingly, these interventions are not expected to produce equivalent effects on *Giardia* infection.

Our findings also demonstrate that antibiotic-mediated microbiome disruption profoundly reshapes the jejunal bile acid milieu, likely reflecting depletion of microbial subpopulations that express bile salt hydrolases (BSHs), the enzymes responsible for bile acid metabolism [94,95]. Although BSH activity was not measured directly, it is expected that reduced availability of unconjugated bile acids would limit subsequent microbial conversion into secondary bile acids, thereby altering the local bile acid composition. Consistently, antibiotic treatment significantly reduced major jejunal primary and secondary bile acid pools and abolished the protective phenotype associated with bile acid sequestration or myeloid FXR deficiency.

Given the well-established roles played by bile acids in regulating both intestinal mucosal immunity and *Giardia* biology, these microbiome-dependent changes in bile acid composition may contribute to the impaired host resistance observed following antibiotic treatment.

## Conclusions

Our findings in the current study suggest that bile acid sequestration increased the diversity and richness of the gut microbiome and contributed to increased resistance to *Giardia* infection in mice fed cholestyramine. While some of the observed changes between the cohorts could be attributed to age, facility drift, and diet-related parameters, these findings suggest that bile acids may function as limiting factors in restricting the diversity and richness of microbial ecosystems in the intestinal tract.

Bile acids can support trophozoite growth, potentially explaining why *Giardia* trophozoites tend to primarily colonize the upper portions of the small intestine where bile acids are abundantly found [96,97]. *G. duodenalis* trophozoites can absorb and internalize conjugated bile acids in the cytosol in a concentration-dependent and carrier-mediated manner *in vitro* [98,99]. In addition to supporting trophozoite growth, bile acids also serve as critical environmental cues for encystation in the distal small intestine, thereby facilitating stage conversion and maintaining completion of the parasite life cycle [100,101]. While these observations suggest that bile acid sequestration could directly influence *Giardia* biology, our findings indicate that cholestyramine-mediated microbiome remodeling is likely the predominant mechanism underlying enhanced resistance to infection. This is supported by the comparable parasite burdens observed in *G. duodenalis*-infected mice (Fig 2C and 2D) and during the later stages of *G. muris* infection (Fig 2B).

Collectively, a better understanding of the intricate interactions between bile acids, *Giardia* parasites, and the gut microbiome should enable the development of novel therapeutic strategies to manipulate intestinal microbial diversity to enhance resistance to enteric microbial infection and improve mucosal vaccine efficacy in human giardiasis.

### Ethics approval and consent to participate

Laboratory animal studies were performed at the Center for Biomedical Research at the University of North Dakota in compliance with procedures approved by the Institutional Animal Care and Use Committee (protocol numbers: 2007–2, 2008–6, 2308–0049, 2310–0059) of the University of North Dakota in accordance with the National Institutes of Health Guidelines.

## Supporting information

S1 Fig Targeted UPLC-MS/MS analysis of bile acid concentrations in the mid-jejunum of *Giardia*-infected C57BL/6 mice (day 7) receiving a control (*n* = 7) or cholestyramine diet (*n* = 6). The bile acid concentrations in the jejunum (ng/g tissue) were expressed as mean ± SEM. *n.s*., not significant, as determined by Mann-Whitney *U* test.
(TIF)

S2 Fig Weight change dynamics in age- and sex-matched mice fed a control or cholestyramine diet after *G. muris* infection. Data were expressed as mean ± SEM of *n* = 6/group. *n.s*., not significant, as determined by Mann-Whitney *U* test. Data are pooled from two independent experiments.
(TIF)

S3 Fig (A) Expression of *Fcgrt*, *Fcgr1*, *Fcgr2b*, and *Fcgr4* in the jejunum of C57BL/6 mice fed a control or (B) cholestyramine diet in uninfected mice as well as in *G. muris*-infected mice 7 and 14 days after infection, as demonstrated by NanoString. Data were expressed as mean ± SEM. *n.s*., not significant, as determined by a one-way ANOVA, followed by Tukey’s post-hoc adjustment for multiple comparisons.
(TIF)

S4 Fig (A) Expression of genes responsible for the biogenesis of prostaglandins, *Ptger2*, *Ptger4*, and *Ptgs2*, in the jejunum of C57BL/6 mice fed a control or (B) cholestyramine diet in uninfected mice as well as in *G. muris*-infected mice 7 and 14 days after infection, as demonstrated by NanoString. Data were expressed as mean ± SEM. *n.s*., not significant, as determined by a one-way ANOVA, followed by Tukey’s post-hoc adjustment for multiple comparisons.
(TIF)

S5 Fig Uncropped Western blot images used to generate Fig 4C. LPS, lipopolysaccharide; CDCA, chenodeoxycholic acid (a natural FXR agonist).
(TIF)

S6 Fig Uncropped Western blot images used to generate Fig 4F. LPS, lipopolysaccharide; LCA, lithocholic acid (a natural TGR5 agonist).
(TIF)

## Abbreviations

AA: Arachidonic acid
ALOX5: Arachidonate 5-lipoxygenase
ALOX5AP: Arachidonate 5-lipoxygenase activating protein
BMDM: Bone marrow-derived macrophage
BSH: Bile salt hydrolase
CFU: Colony-forming unit
COX2: Cyclooxygenase-2
CPA3: Carboxypeptidase A3
CYP7A1: Cholesterol 7α-hydroxylase
EDTA: Ethylenediaminetetraacetic acid
ELISA: Enzyme-linked immunosorbent assay
Fcer1a: Fc epsilon receptor Ia
FGF19: Fibroblast growth factor 19
FXR: Farnesoid X receptor
Gpbar1: G protein-coupled bile acid receptor 1
H&E: Hematoxylin and eosin
HETE: Hydroxyeicosatetraenoic acid
IEC: Intestinal epithelial cell
LP: Lamina propria
LDL: Low-density lipoprotein
MLN: Mesenteric lymph node
Nr1h4: Nuclear receptor subfamily 1 group H member 4
OD: Optical density
PCA: Principal component analysis
Ptger: Prostaglandin E receptor
TGR5: Takeda G protein-coupled receptor
T_H2_: T helper 2
T_H17_: T helper 17
TSA: Tryptic soy agar
UPLC-MS/MS: Ultra-performance liquid chromatography-tandem mass spectrometry.
